# Supplementary material for: Male mating strategies to counter sexual conflict in spiders
Source: Commun Biol. 2022 Jun 2;5:534. doi: 10.1038/s42003-022-03512-8 (PMC9163124; doi:10.1038/s42003-022-03512-8)
Supplement: Supplementary file 2 — Supplementary information [file 42003_2022_3512_MOESM2_ESM.pdf]

Male mating strategies to counter sexual conflict in spiders

Supplementary Information

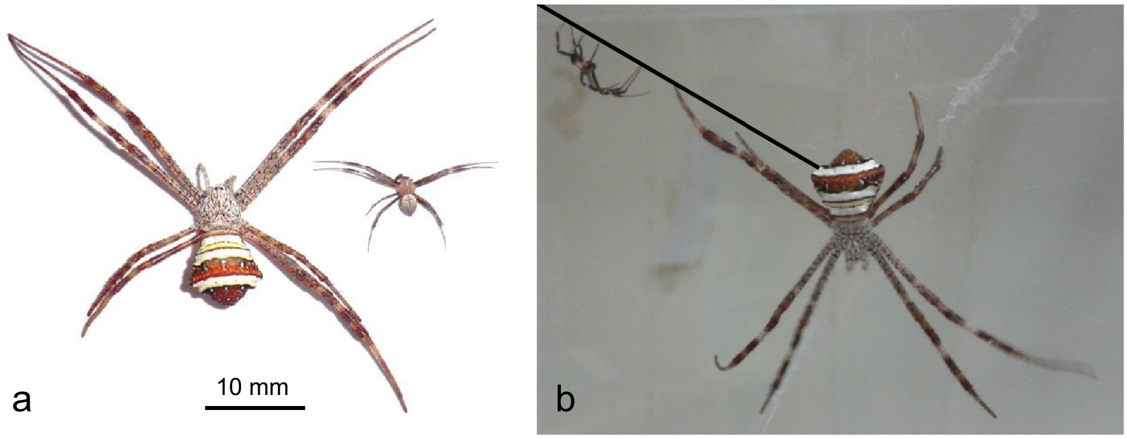

Supplementary Fig. 1 ***Argiope versicolor*****. a** Sexual dimorphism between female (left) and male (right). **b** Vibrational courtship by male on mating thread (depicted by black line inserted).

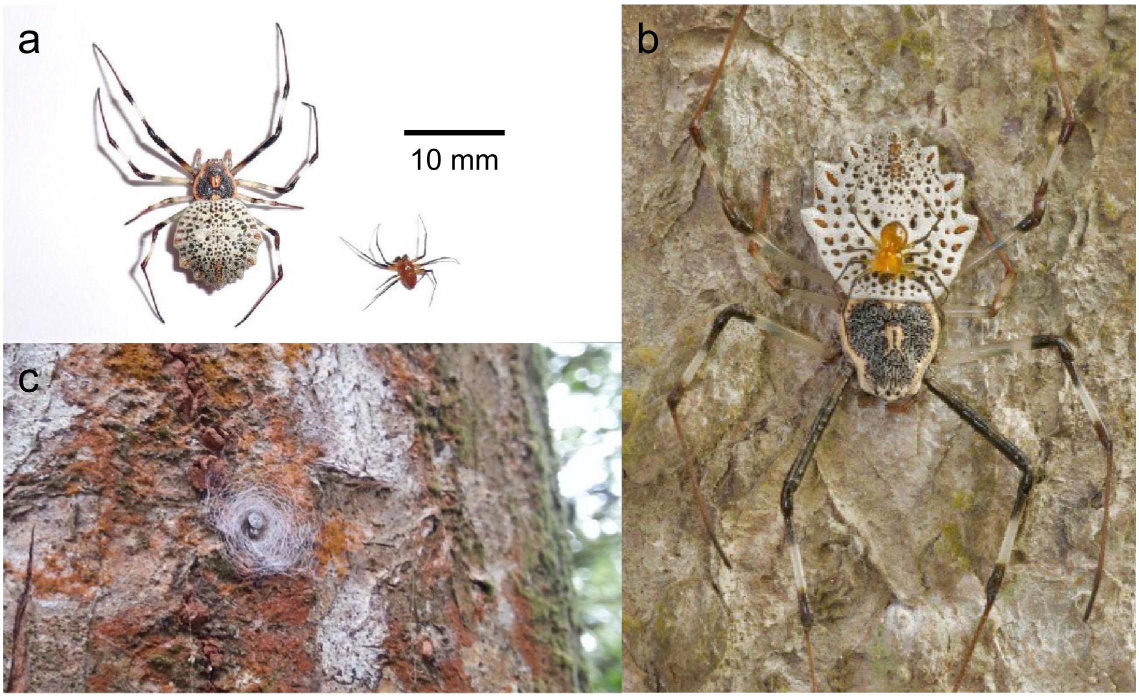

Supplementary Fig. 2 ***Herennia multipuncta*****. a** Sexual dimorphism between female (left) and male (right). **b** A male awaits for female consent for copulation, i.e. when she lifts her abdomen. **c** the epigynum of the female is blocked as she positions herself in the web's central area termed the hub-cup.

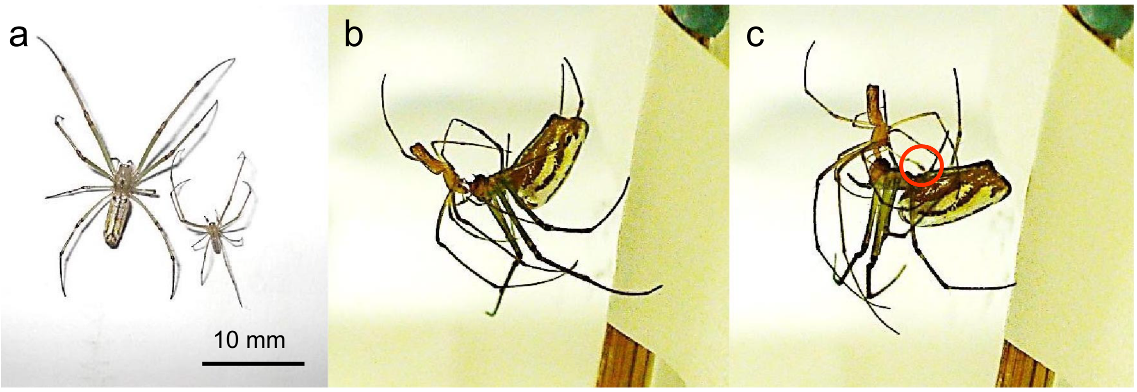

Supplementary Fig. 3 ***Leucauge decorata*****. a** Sexual dimorphism between female (left) and male (right). **b** During copulation, the pairs exhibit chelicerae clasping. **c** The male tries to insert his stretched palp (circled) after female is visibly calm and relaxed (her legs are drooping down).

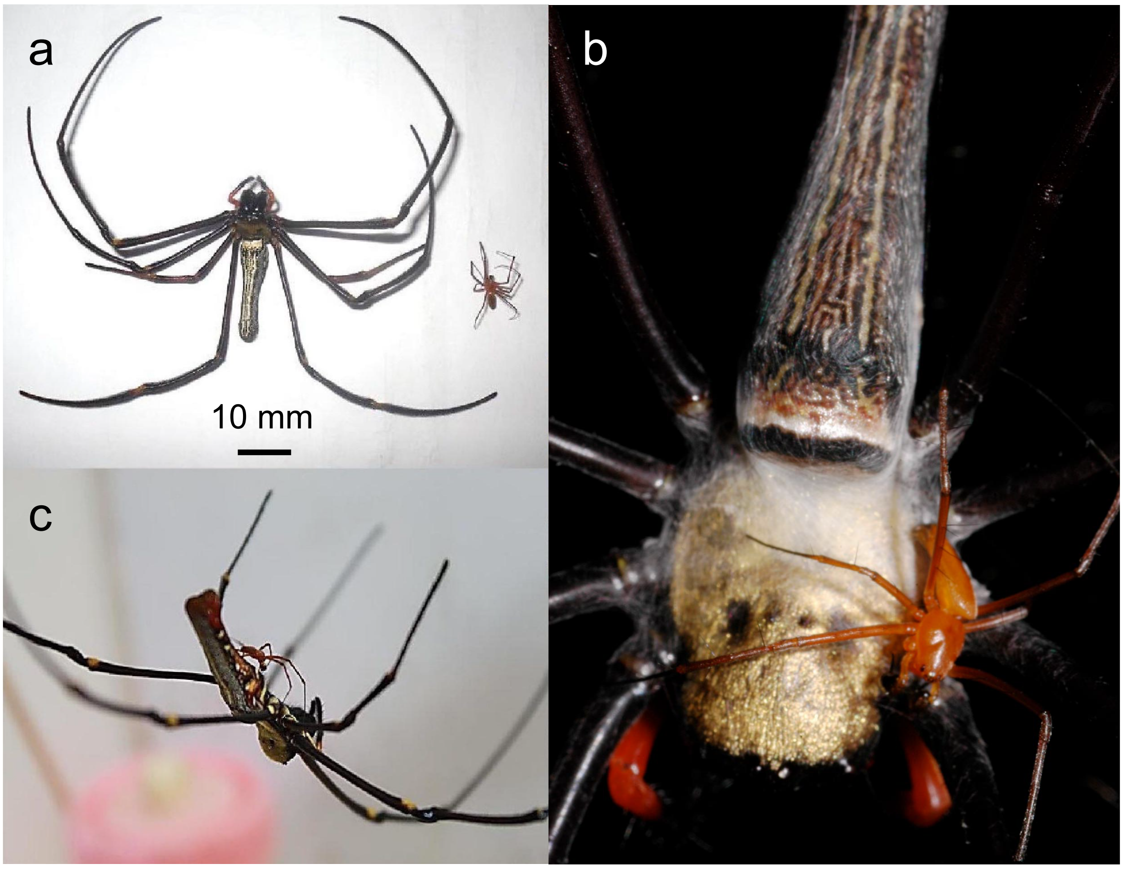

Supplementary Fig. 4 ***Nephila pilipes*****. a** Sexual dimorphism between female (left) and male (right). **b** Mate binding during courtship on the female's cephalothorax, abdomen and legs. **c** Male inserting his palp into female's epigynum during copulation.

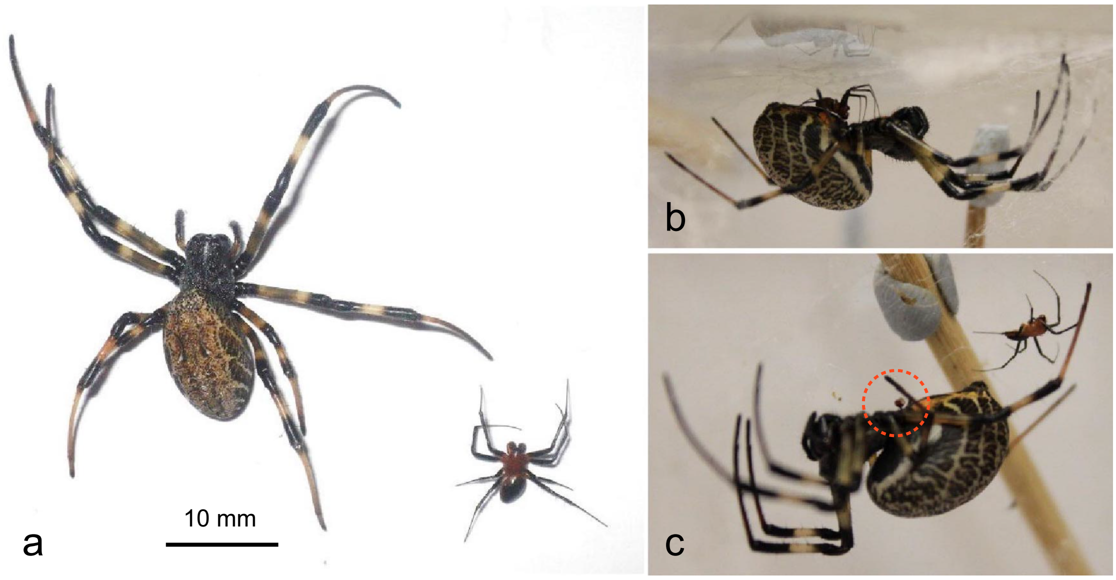

Supplementary Fig. 5 *Nephilengys malabarensis*. a Sexual dimorphism between female (left) and male (right). **b** A male on top of the female to insert his palp during copulation. **c** A male breaks off his palp which is lodged in the female's epigynum (circled).

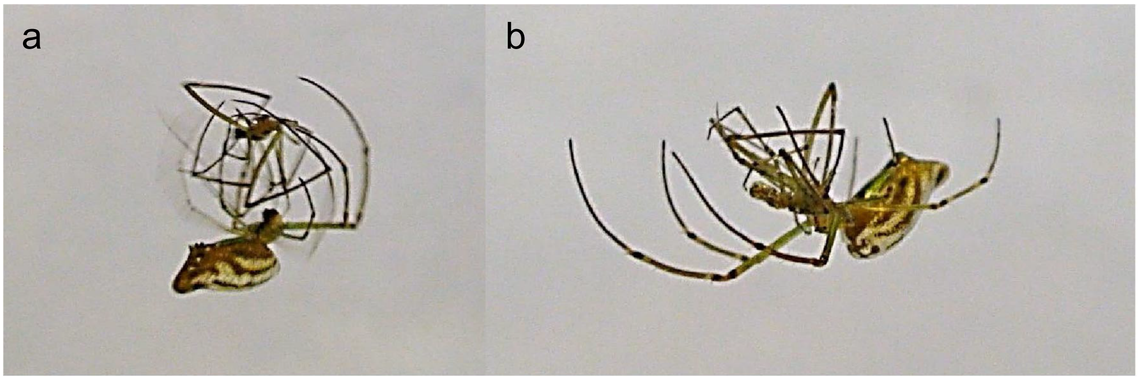

Supplementary Fig. 6 A female *Leucauge decorata* cannibalizing her male partner pre-insemination. a After grabbing the male, the female spins silk around him. **b** Killing the male and injecting her venom.

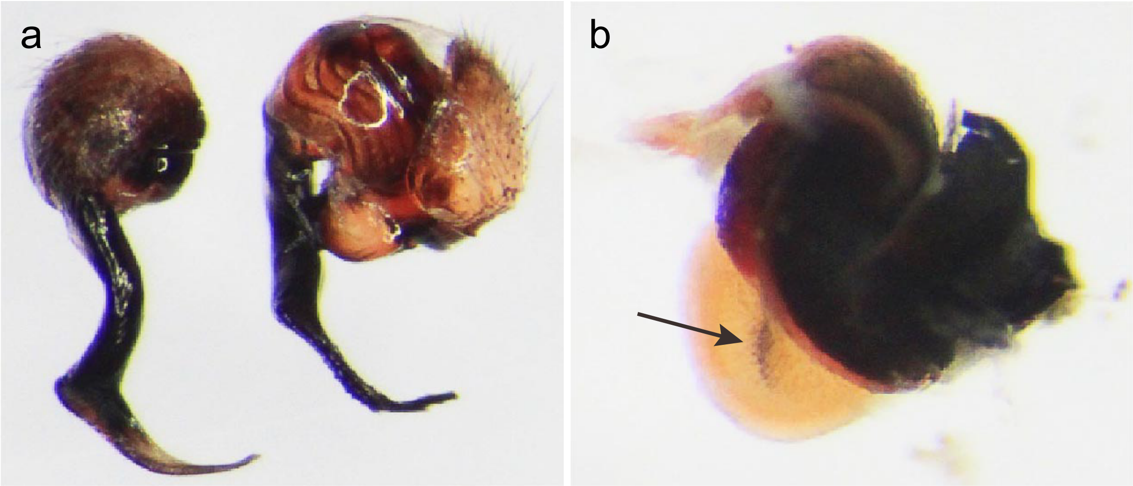

Supplementary Fig. 7 Palp mutilation – partial breakage. a Tip of embolus is broken off (on the right) after the palp is used for copulation in *Nephilengys malabarensis*. **b** A possible embolus breakage lodged in the female's spermatheca of *Nephilengys malabarensis* (shown by arrow).

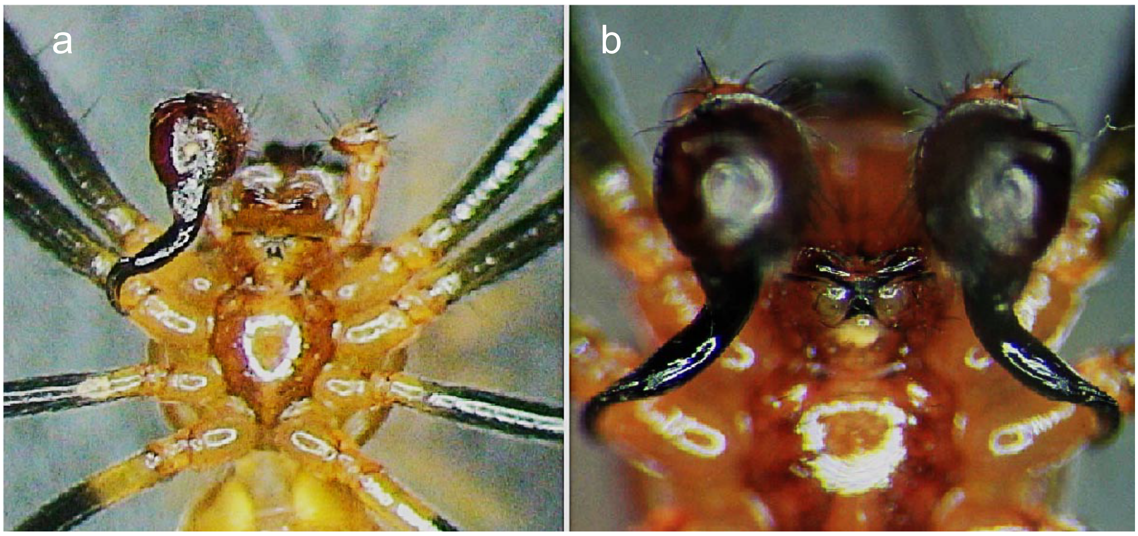

Supplementary Fig. 8 Male *Nephilengys malabarensis* showing palps. a A half eunuch with one broken palp. **b** A male with both palps intact.

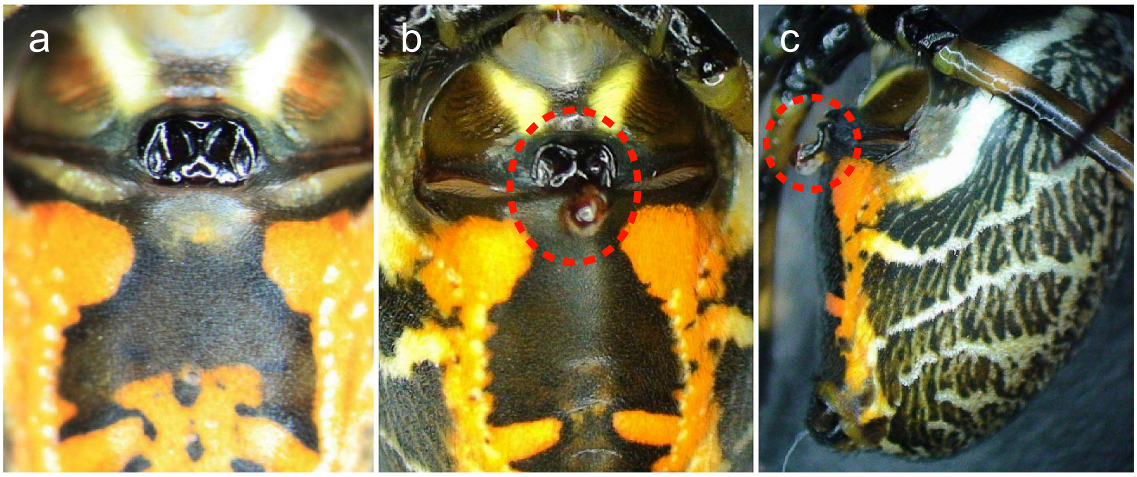

Supplementary Fig. 9 Female *Nephilengys malabarensis* showing epigyna.
a A clear epigynum without palp. **b** Ventral view of a male's broken palp lodged in the epigynum (circled). **c** Lateral view of a male's broken palp lodged in the epigynum (circled).

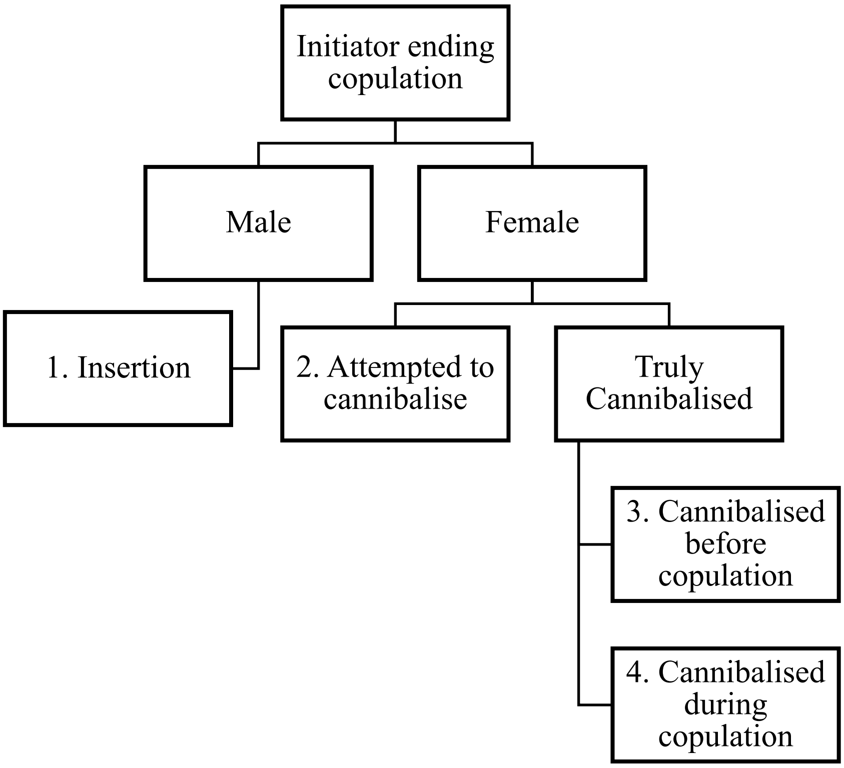

Supplementary Fig. 10 A flowchart showing the way of categorising the outcomes of copulations.

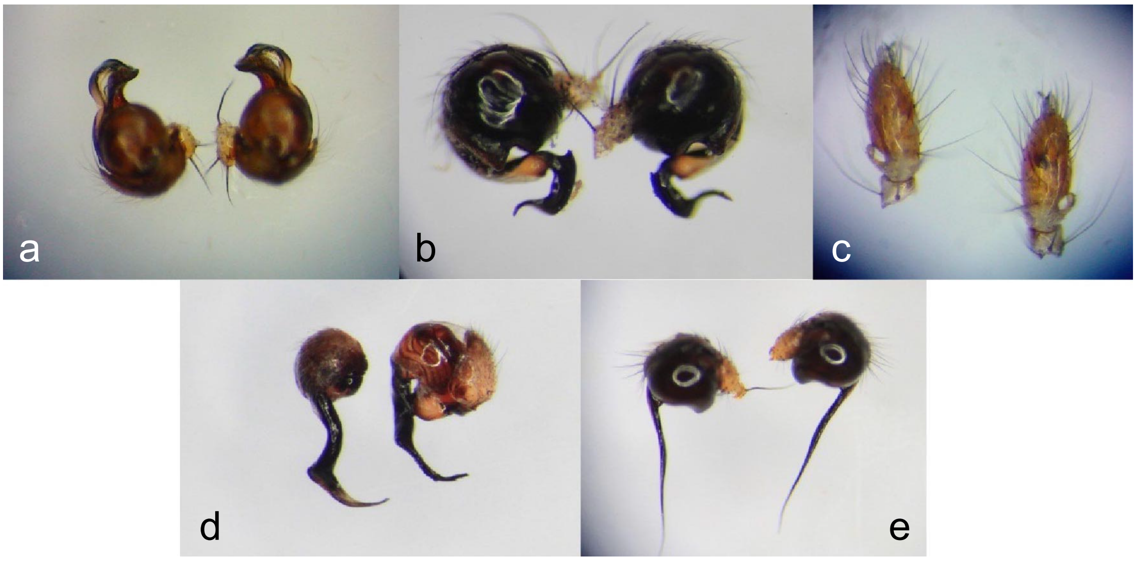

Supplementary Fig. 11 Palp detachments. a *Argiope versicolor.* **b** *Herennia multipuncta*. **c** *Leucauge decorate* **d** *Nephilengys malabarensis*. **e** *Nephila pilipes*.

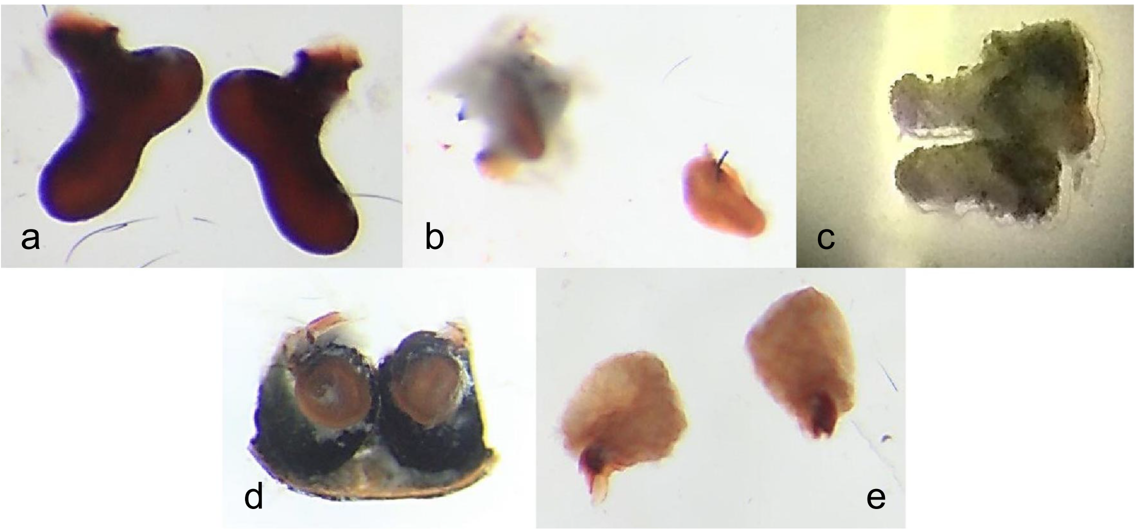

Supplementary Fig. 12 Paired spermatheca of female epigynum. a *Argiope versicolor.* **B**. *Herennia multipuncta* spermathecae unscraped and covered with tissues (left) and one that has been scraped off tissues (right). **c** *Leucauge decorate*. **d** *Nephilengys malabarensis*. **e** *Nephila pilipes*.

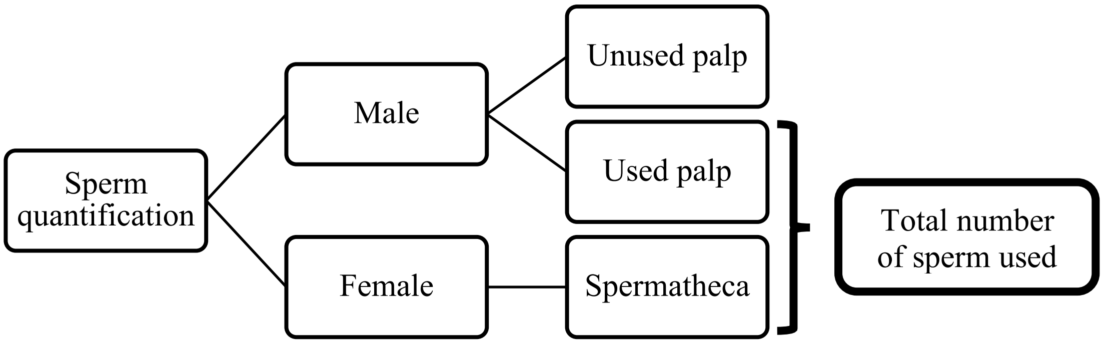

Supplementary Fig. 13 A flowchart showing the method of sperm counting.

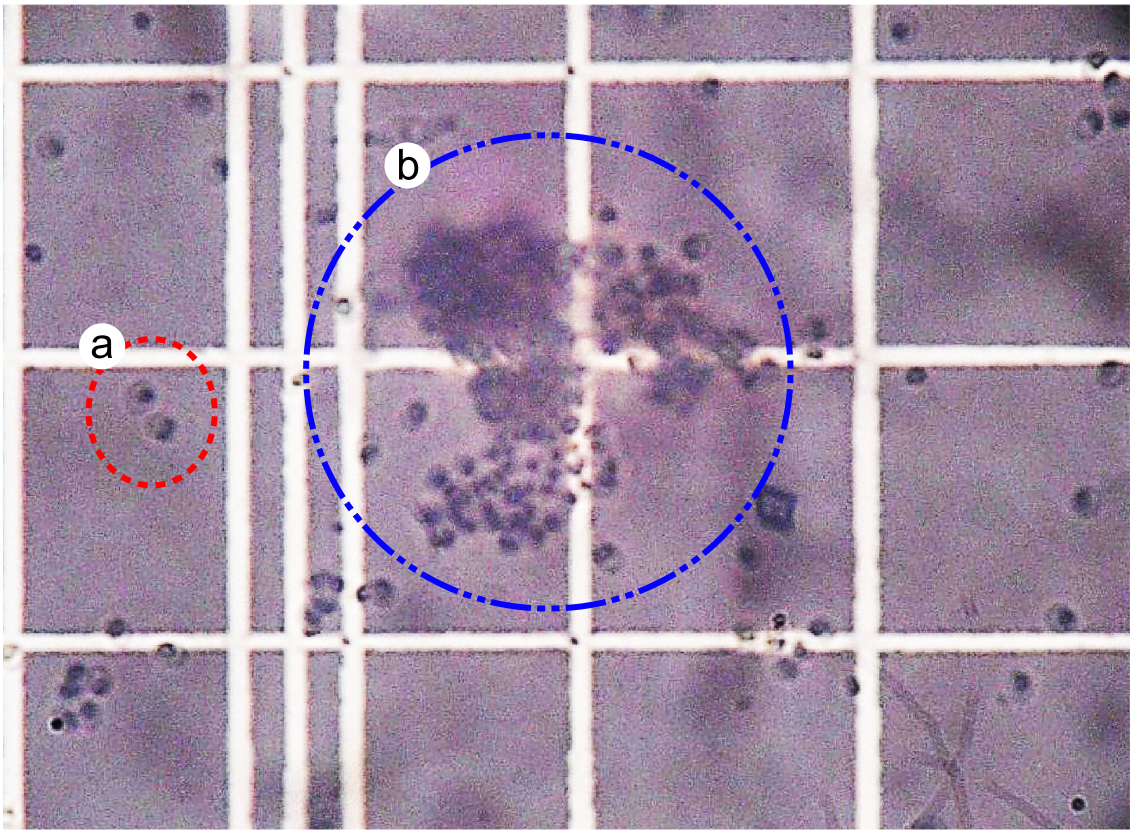

Supplementary Fig. 14 Sperm seen from a haemocytometer under a compound microscope at 400× magnification. a Individual sperm capsules. **b** Sperm clumps which make counting difficult.

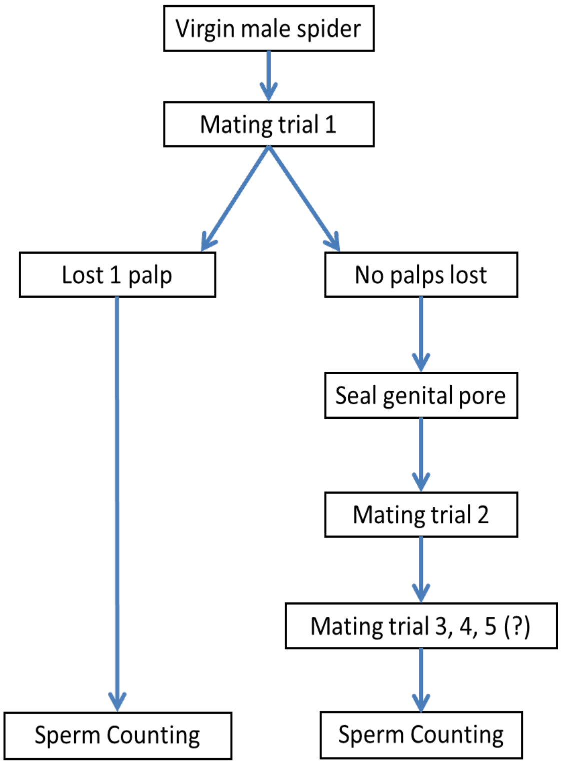

Supplementary Fig. 15 Flowchart summary of procedures for different groups of male spiders.

Supplementary Table 1 Orb-weaving spider species with the rate (%) of sexual cannibalism according to two definitions of sexual cannibalism. The restricted definition was used in this study.

Species	Broad definition (aggressive, grabbed, attacked, or killed) (%)	Restricted definition (grabbed or killed) (%)	Rank
Argiope versicolor	26.3	26.3	1
Herennia multipuncta	36.4	9.1	4
Leucauge decorata	35.3	11.8	3
Nephilengys malabarensis	32.1	21.4	2
Nephila pilipes	66.7	0	5

Supplementary Table 2 Comparison of 26 generalized linear models to predict the effects of spider family, species, female cannibalism (FC), sexual size dimorphism (SSD), female post-maturity age (FA), male post-maturity age (MA) on whether males used the palp with more sperm for their first insertion (in terms of the ratio of the number of sperm of the used palp for the first insertion to that of the unused palp).

Predictors	*df*	LogLik	AICc	Delta	Weight
Family+species+FC+SSD+FA+MA+FC:FA+FC:MA	12	-111.5	252.1	0	0.788
Family+species+FC+SSD+FA+MA+FC:SSD+FC:FA+FC:MA	13	-111.4	255.1	2.9	0.183
Family+species+FC+SSD+FA+MA+FC:SSD+FC:MA	12	-115.5	260.1	8.0	0.014
Family+species+FC+SSD+FA+MA	10	-119.2	262.0	9.9	0.006
Family+species+FC+SSD+FA+MA+FC:SSD+FC:FA	12	-116.6	262.4	10.3	0.005
Family+species+FC+SSD+FA	9	-121.7	264.2	12.0	0.002
Family+species+FC+SSD+FA+MA+FC:SSD	11	-119.1	264.6	12.4	0.002
Family+species+FC+SSD	8	-126.2	270.7	18.6	0
Family+species+SSD	3	-132.8	271.9	19.7	0
FC+SSD	4	-132.2	273.0	20.9	0
FC+SSD+FC:SSD	5	-131.6	274.1	21.9	0
FC:SSD	4	-132.7	274.1	21.9	0
Family+species	6	-138.9	290.9	38.8	0
Species	6	-138.9	290.9	38.8	0
FA	3	-143.1	292.5	40.4	0
FC:FA	4	-142.1	292.7	40.6	0
Family+species+FC	7	-138.6	292.7	40.6	0
FC+FA+MA+FC:FA	5	-141.2	293.2	41.0	0
FC+ FA	4	-143.0	294.5	42.4	0
Null ~ 1	2	-148.9	301.9	49.8	0
MA	3	-148.4	303.0	50.9	0
FC	3	-148.5	303.4	51.2	0
Family	3	-148.9	304.1	51.9	0
FC+MA	4	-148.1	304.6	52.5	0
FC:MA	4	-148.4	305.2	53.1	0
FC+MA+FC:MA	5	-147.6	306.0	53.8	0

Supplementary Table 3 Comparison of 26 generalized linear models to predict the effects of spider family, species, female cannibalism (FC), sexual size dimorphism (SSD), female post-maturity age (FA), male post-maturity age (MA), and copulation duration (CD) on the percentage (%) of the sperm transferred.

Predictors	*df*	LogLik	AICc	Delta	Weight
Family+species+FC+SSD+FA+MA+CD+FC:SSD+FC:FA+ FC:MA+FC:CD	15	-293.4	625.2	0	0.301
Family+species+FC+SSD+FA+MA+FC:SSD+FC:FA	12	-298.4	626.1	0.9	0.197
Family+species+FC+SSD	8	-304.0	626.3	1.1	0.178
Family+species+FC+SSD+FA	9	-303.2	627.4	2.1	0.104
Family+species+FC+SSD+FA+MA+FC:SSD	11	-300.9	628.2	3.0	0.069
Family+species+FC+SSD+FA+MA+FC:SSD+FC:MA	12	-300.0	629.1	3.9	0.043
Family+species+FC+SSD+FA+MA	10	-303.2	629.9	4.6	0.030
FC+SSD	4	-310.8	630.1	4.9	0.027
FC:SSD	4	-310.9	630.4	5.2	0.023
FC+SSD+FC:SSD	5	-310.1	631.2	5.9	0.016
Family+species+FC+SSD+FA+MA+FC:FA+FC:MA	12	-301.2	631.7	6.5	0.012
SSD	3	-316.6	639.5	14.3	0
FC:MA	4	-379.4	767.2	142.0	0
FC	3	-380.6	767.5	142.2	0
FC+FA	4	-380.3	769.2	144.0	0
FC+MA	4	-380.4	769.4	144.1	0
FC+MA+FC:MA	5	-379.3	769.4	144.2	0
FC+FA+FC:FA	5	-379.9	770.5	145.2	0
Null ~ 1	2	-383.8	771.8	146.5	0
FC:FA	4	-382.2	772.9	147.7	0
Family+species+FC	7	-378.8	773.0	147.8	0
Family	3	-383.7	773.7	148.5	0
FA	3	-383.7	773.8	148.5	0
MA	3	-383.8	773.8	148.6	0
Family+species	6	-383.1	779.3	154.0	0
Species	6	-383.1	779.3	154.0	0

Supplementary Table 4 Comparison of seven generalized linear mixed-effects models (GLMM) to predict the effects of sexual size dimorphism (SSD), positive difference in sperm number between the used and unused palps, sperm ratio of the used palp to the unused palp and palp breakage on palp choice.

Predictors	*df*	LogLik	AICc	Delta	Weight
Log(positive difference)+log(sperm ratio)+log(SSD)+palp breakage+(1|maleID)	7	-11.3	48.9	0.0	0.840
Log(positive difference)+log(sperm ratio)+log(SSD)+(1|maleID)	6	-18.3	53.1	4.1	0.106
1+(1|maleID)	3	-24.0	54.8	5.9	0.044
Log(sperm ratio)+(1|maleID)	4	-24.6	58.6	9.7	0.007
Log(positive difference) +(1|maleID)	4	-25.9	61.2	12.3	0.002
Log(positive difference):log(sperm ratio)+(1|maleID)	4	-27.1	63.5	14.6	0.001
Log(positive difference)+log(sperm ratio)+(1|maleID)	5	-26.1	64.4	15.4	0
