# Supplementary material for: Male mating strategies to counter sexual conflict in spiders
Source: Commun Biol. 2022 Jun 2;5:534. doi: 10.1038/s42003-022-03512-8 (PMC9163124; doi:10.1038/s42003-022-03512-8)
Supplement: Supplementary file 3 — Description of Additional Supplementary Files [file 42003_2022_3512_MOESM3_ESM.pdf]

Description of Additional Supplementary Files

File name: Supplementary Data 1
Description: The source data behind the graphs presented in the Figs. 1, 2, 3.
