# Supplementary material for: Male mating strategies to counter sexual conflict in spiders
Source: Commun Biol. 2022 Jun 2;5:534. doi: 10.1038/s42003-022-03512-8 (PMC9163124; doi:10.1038/s42003-022-03512-8)
Supplement: Supplementary file 5 — Reporting Summary [file 42003_2022_3512_MOESM5_ESM.pdf]

nature portfolio

Corresponding author(s): Daiqin Li
Matjaž Kuntner

Last updated by author(s): Apr 9, 2022

Reporting Summary

Nature Portfolio wishes to improve the reproducibility of the work that we publish. This form provides structure for consistency and transparency in reporting. For further information on Nature Portfolio policies, see our Editorial Policies and the Editorial Policy Checklist.

Statistics

For all statistical analyses, confirm that the following items are present in the figure legend, table legend, main text, or Methods section.

n/a	Confirmed	
☐	☒	The exact sample size (*n*) for each experimental group/condition, given as a discrete number and unit of measurement
☐	☒	A statement on whether measurements were taken from distinct samples or whether the same sample was measured repeatedly
☐	☒	The statistical test(s) used AND whether they are one- or two-sided<br>*Only common tests should be described solely by name; describe more complex techniques in the Methods section.*
☐	☒	A description of all covariates tested
☐	☒	A description of any assumptions or corrections, such as tests of normality and adjustment for multiple comparisons
☐	☒	A full description of the statistical parameters including central tendency (e.g. means) or other basic estimates (e.g. regression coefficient) AND variation (e.g. standard deviation) or associated estimates of uncertainty (e.g. confidence intervals)
☐	☒	For null hypothesis testing, the test statistic (e.g. *F*, *t*, *r*) with confidence intervals, effect sizes, degrees of freedom and *P* value noted<br>*Give P values as exact values whenever suitable.*
☒	☐	For Bayesian analysis, information on the choice of priors and Markov chain Monte Carlo settings
☒	☐	For hierarchical and complex designs, identification of the appropriate level for tests and full reporting of outcomes
☒	☐	Estimates of effect sizes (e.g. Cohen's *d*, Pearson's *r*), indicating how they were calculated

Our web collection on statistics for biologists contains articles on many of the points above.

Software and code

Policy information about availability of computer code

Data collection	No special software was used.
Data analysis	All statistical analyses and figures were done using R 4.1.1 (R Core Team: a language and environment for statistical computing. Vienna, Austria: R Foundation for Statistical Computing. Retrieved from http://www.R-project.org. (2021)).

For manuscripts utilizing custom algorithms or software that are central to the research but not yet described in published literature, software must be made available to editors and reviewers. We strongly encourage code deposition in a community repository (e.g. GitHub). See the Nature Portfolio guidelines for submitting code & software for further information.

Data

Policy information about availability of data

All manuscripts must include a data availability statement. This statement should provide the following information, where applicable:
- Accession codes, unique identifiers, or web links for publicly available datasets
- A description of any restrictions on data availability
- For clinical datasets or third party data, please ensure that the statement adheres to our policy

All data that support the findings of this study and all code required to reproduce the analyses of the data are available from the Zenodo:

Field-specific reporting

Please select the one below that is the best fit for your research. If you are not sure, read the appropriate sections before making your selection.

☐ Life sciences ☐ Behavioural & social sciences ☒ Ecological, evolutionary & environmental sciences

For a reference copy of the document with all sections, see nature.com/documents/nr-reporting-summary-flat.pdf

Ecological, evolutionary & environmental sciences study design

All studies must disclose on these points even when the disclosure is negative.

Study description	When sexual conflict selects for reproductive strategies that only benefit one of the sexes, evolutionary arms races may ensue. Female sexual cannibalism is an extreme manifestation of sexual conflict. Here we test two male mating strategies aiming at countering sexual cannibalism in spiders. The “better charged palp” hypothesis predicts male selected use of the paired sexual organ (palp) containing more sperm for their first copulation. The “fast sperm transfer” hypothesis predicts accelerated insemination when cannibalism is high. Our comparative tests on five orbweb spider species with varying levels of female sexual cannibalism and sexual size dimorphism (SSD) reveal that males choose the palp with more sperm for the first copulation with cannibalistic females and that males transfer significantly more sperm if females are cannibalistic or when SSD is biased. By supporting the two hypotheses, these results provide credibility for male mating syndrome. They, however, open new questions, namely, how does a male differentiate sperm quantities between his palps? How does he perform palp choice after assessing his cannibalistic partner? By conducting follow-up experiments on Nephilengys malabarensis, we reveal that it is sperm volume detection, rather than left-right palp dominance, that plays prominently in male palp choice. We used 5 species of orbweb spiders with different levels of sexual cannibalism and sexual size dimorphism, which were the two main factors. Male and female post-maturation ages were also included as factors. In the statistical models, we included these 4 factors and their 2-way interactions. A total 86 of successful mating trials were conducted for five species in the comparative study and 34 mating trials in the palp choice mechanism part. Each mating trial represents a replicate.
Research sample	Since we tested the hypotheses that the two male mating strategies (palp choice and sperm transfer rate adjustment) vary with the levels of female sexual cannibalism and/or sexual size dimorphism, we chose five orbweb spider species from two families: Argiope versicolor (Araneidae), Herennia multipuncta, Nephila pilipes, and Nephilengys malabarensis (Araneidae), and Leucauge decorata (Tetragnathidae) to represent gradients in sexual cannibalism and sexual size dimorphism. Since reproductive status (mated or not) would affect the mating results, we thus collected large juvenile spiders and reared them until they became adults to ensure that they were all virgin. We then used only virgin males and females for all the mating trials without any manipulation. We collected spiders from a few localities for each species in Singapore, which is a small island, thus we thought these should represent the population of each species in Singapore. The replicates are individual spiders for each species.
Sampling strategy	No sample size calculation was performed as in tropics the abundance of spiders of each species was not very high compared to spider species in temperate. We tried our best to collect the numbers of spiders for each species available in the field during the period of this study. Though we would like to have more spiders, but the number of spiders for each species were what I could be collected. Thus, the sample size was not balanced among species that were used in this study.
Data collection	Noeleen Tan, Xaven Wong, Shichang Zhang under the supervision of Daiqin Li and Matjaz Kuntner conducted experiments. Data was recorded directly into notebooks and/or Excel spreadsheets.
Timing and spatial scale	All experiments were conducted in the laboratory. We started mating trials immediately after spiders matured and counted sperms once the mating was finished.
Data exclusions	No data were excluded.
Reproducibility	All attempts to repeat experiments were successful. The methods are detailed and should be reproducible by someone experienced with spiders. The very important sperm counting is very detailed and reproducible.
Randomization	In all mating trials in each species, a male and female were randomly selected and paired. For sperm counting, We extracted two 4 µl samples using a pipette and placed them on both chambers of a 0.1 mm Neubauer improved haemocytometer (Blaubrand, Wertheim, Germany). Using a compound microscope at 400× magnification, we counted sperm in all 25 quadrats (0.05 × 0.05 mm) from each chamber. As only encapsulated sperm could be observed under the microscope, we equated the number of capsules to the number of sperm. An average was calculated from the quantities in the two randomly selected chambers, which was then multiplied by the dilution factor to get the total number of sperm of the sample.
Blinding	No blinding was used in these experiments. The mating trials and sperm counting are not subjective here.

Did the study involve field work? ☒ Yes ☐ No

Field work, collection and transport

Field conditions	Singapore, tropics. Spiders were commonly found in the secondary forests or shrubs. We collected spiders when it didn't rain.
Location	There are a multitude of collection sites in Singapore. We could provide the Lat/Long coordinates for each locality if useful.

Access & import/export	The spiders were found in secondary forests or shrubs in Singapore. We had the permits from Singapore National Parks. All spiders were collected and studied in Singapore, thus there is no import/export of these spiders for this study.
Disturbance	No disturbance in the field during or after having removing the spiders from the sites.

Reporting for specific materials, systems and methods

We require information from authors about some types of materials, experimental systems and methods used in many studies. Here, indicate whether each material, system or method listed is relevant to your study. If you are not sure if a list item applies to your research, read the appropriate section before selecting a response.

Materials & experimental systems

n/a	Involved in the study	
☒	☐	Antibodies
☒	☐	Eukaryotic cell lines
☒	☐	Palaeontology and archaeology
☐	☒	Animals and other organisms
☒	☐	Human research participants
☒	☐	Clinical data
☒	☐	Dual use research of concern

Methods

n/a	Involved in the study	
☒	☐	ChIP-seq
☒	☐	Flow cytometry
☒	☐	MRI-based neuroimaging

Animals and other organisms

Policy information about studies involving animals; ARRIVE guidelines recommended for reporting animal research

Laboratory animals	The animals we work with are spiders. There are no standards in place for spiders; however, our spiders were well cared for using established protocols.
Wild animals	Argiope versicolor (Doleschall, 1859), Herennia multipuncta (Doleschall, 1859), Leucauge decorata (Blackwall, 1864), Nephila pilipes (Fabricius, 1793) and Nephilengys malabarensis (Walckenaer, 1841) ; sample size: A. versicolor: N = 18; H. multipuncta: N = 11; L. decorata: N = 17; N. malabarensis: N = 28 in comparative study and N = 23 males and N = 34 females in palp choice mechanism study; N. pilipes: N = 12; we collected them by hand collection using vials. We took spiders back by taxi; We released the spiders to Singapore after the experiments if they were alive. If spiders killed by females during the experiments, we did preserved them. If the spiders died after the experiments, we preserved in 75% ethanol.
Field-collected samples	Spiders were maintained using long established protocols.
Ethics oversight	Approval is not required for spiders at this time.

Note that full information on the approval of the study protocol must also be provided in the manuscript.
